# Body-technology interfaces: digital corporeality and emerging psychopathology

**DOI:** 10.3389/fpsyt.2026.1740203

**Published:** 2026-07-03

**Authors:** Valerio Ricci, Giovanni Martinotti, Stefania Chiappini

**Affiliations:** 1 Azienda Ospedaliero Universitaria San Luigi Gonzaga, Orbassano, Italy; 2 Universita degli Studi Gabriele d’Annunzio Chieti Pescara, Chieti, Italy; 3 Saint Camillus International University of Health and Medical Sciences, Rome, Italy

**Keywords:** body image, digital technology, embodiment, phenomenology, psychopathology, virtual reality

## Abstract

**Background:**

The proliferation of body-technology interfaces — including wearable devices, virtual reality, augmented reality, and digital body modification applications — has significantly influenced contemporary embodied experience, raising novel questions for psychopathology research and clinical practice.

**Objectives:**

This analysis examines how modern body-technology interfaces influence bodily experience and identifies proposed clinical presentations arising from these interactions, discussing their theoretical grounding and implications for assessment and treatment.

**Methods:**

We conducted a comprehensive interdisciplinary review integrating phenomenological, neuroscientific, and clinical perspectives to characterize technology-related embodiment presentations and discuss existing and potential therapeutic approaches within established clinical frameworks. Literature was identified through PubMed/MEDLINE, PsycINFO, and Google Scholar searches (1990–2025), using terms related to embodiment, body-technology interfaces, virtual and augmented reality, wearable devices, and emerging psychopathology. Sources spanning neuroscientific, clinical, phenomenological, and ethnographic perspectives were selected based on direct relevance to the review scope.

**Results:**

Eight proposed clinical constructs are described across four domains: body image disorders (filter dysmorphia, tracking dysperception), embodiment disorders in virtual environments (virtual depersonalization disorder, avatar-body identity conflict), disorders of technologically mediated proprioception (proprioceptive lag syndrome, augmented sensorimotor confusion disorder), and behavioral addictions (self-tracking addiction, virtual embodiment addiction. These constructs are grounded in documented neurobiological mechanisms involving body representation, interoceptive processing, and reward circuits. Current evidence consists primarily of theoretical elaborations, case reports, and ethnographic observations; epidemiological data remain limited. The proposed constructs are best understood as technology-amplified variants of established conditions or as hypotheses warranting empirical investigation rather than validated independent diagnostic entities.

**Conclusions:**

Body-technology interfaces generate psychopathological presentations that existing clinical frameworks may not fully capture. Future research should focus on empirically validating these proposed constructs, developing standardized assessment instruments, and establishing evidence-based therapeutic protocols. Ethical dimensions, including differential access and algorithmic normalization of bodily experience, require ongoing clinical and policy attention.

## 1 Introduction

The emergence of digital technologies has profoundly altered the way humans experience and relate to their bodies. Wearable devices, virtual reality, augmented reality, and other emerging technologies have created novel interfaces between body and technology, contributing to a redefinition of corporeality itself and raising new questions in the psychopathological domain (1, 2).

This analysis explores how modern body-technology interfaces are influencing bodily experience and what new forms of psychopathology may emerge from these interactions. Corporeality, understood as the lived experience of one’s body (the Leib in phenomenological tradition), has always been an element in understanding psychopathology (3). However, in the digital era, the boundaries between physical body and technology have become increasingly blurred, creating what some theorists define as an “extended” or “augmented” corporeality (4, 5). This technological extension of the body raises crucial questions about the nature of bodily experience, sense of self, and relationship with the surrounding world—all central elements in psychopathological manifestations.

Literature was identified through PubMed/MEDLINE, PsycINFO, and Google Scholar searches (1990–2025), using terms related to embodiment, body-technology interfaces, virtual and augmented reality, wearable devices, and emerging psychopathology. Sources spanning neuroscientific, clinical, phenomenological, and ethnographic perspectives were selected based on direct relevance to the review scope.

## 2 The transformation of bodily experience in the digital era

### 2.1 From the biological body to the technologically mediated body

Contemporary bodily experience is increasingly characterized by technological mediation, marking a shift in how we understand and inhabit our physical selves. The body is no longer perceived solely as a biological entity but has evolved into an interface between biological selfhood and technological environments — a dynamic boundary through which embodied experience is increasingly shaped by digital mediation. While this transformation has deep historical roots, it has been exponentially accelerated in recent decades, altering the phenomenology of embodied existence (6).

This evolution builds upon earlier anthropological insights. Marcel Mauss, in his seminal work “Techniques of the Body” (1936), had already recognized how bodily techniques were culturally determined and learned rather than purely biological. Today, we witness an unprecedented expansion of this principle as devices such as smartphones, smartwatches, and other wearable technologies introduce new “techniques of the body” that modify not only behaviors but also bodily perception itself (7). These digital extensions represent a qualitative leap from traditional tools, creating what might be understood as a technologically mediated corporeality that challenges assumptions about the boundaries of selfhood.

The incorporation of tools and devices into bodily experience has been documented by neuroscientific and embodied cognition research. Maravita and Iriki (8) demonstrated that tool use induces lasting modifications in the peripersonal space representation of the body schema, suggesting that technological incorporation operates at the level of neural body maps rather than merely behavioral adaptation. Cardinali et al. (9) similarly showed that tool use induces morphological updating of the body schema, with effects persisting after the tool is removed. This process is consistent with Merleau-Ponty (10) phenomenological account of tool incorporation, in which a tool becomes phenomenologically transparent when integrated into skilled bodily engagement — receding from conscious attention as it becomes an extension of intentional action. It is important to distinguish this embodied incorporation from Clark and Chalmers (11) extended mind theory, which concerns the extension of cognitive processes beyond the skull — such as the use of notebooks or external memory aids — and does not address the extension of the lived body or its phenomenological boundaries. The claim that digital devices extend the body should therefore be grounded in embodied cognition research and phenomenology of the body schema, rather than in cognitive extension theory.

The temporal dimension of this transformation is perhaps most evident in how self-tracking devices create new forms of bodily temporality. These technologies enable constant monitoring of bodily parameters, establishing what Rettberg (12) terms a new temporal awareness of the body that differs qualitatively from traditional modes of bodily self-perception. Schüll (13) observes that this creates a novel “temporal architecture” of bodily existence, where the body becomes subject to continuous monitoring and evaluation, transforming the rhythms of embodied life from cyclical and intuitive patterns to linear and quantified progressions.

Spatially, telepresence and virtual reality technologies enable bodily experiences that transcend the traditional limits of physical space, creating what Morie (14) describes as new geographies of embodiment. Research by Slater et al. (15) has demonstrated how virtual embodiment can create authentic sensations of “being there,” challenging not only traditional notions of bodily location but also the relationship between consciousness and spatial presence. It should be noted, however, that this spatial transformation does not imply a severance of embodiment from the physical body: the sense of presence in virtual environments remains grounded in the physical body’s sensorimotor contingencies, representing a context-dependent remapping of self-location rather than genuine disembodiment (16). This spatial extension suggests that embodied presence is no longer necessarily tied to physical co-location, opening possibilities for what might be called distributed embodiment.

The perceptual transformation enabled by human-machine interfaces represents another crucial dimension of this shift. These technologies amplify or modify natural perceptive capabilities, creating hybrid forms of sensing that blur the boundaries between biological and technological perception (17). Studies on sensory substitution devices have revealed remarkable neural plasticity in adapting to technologically mediated perception, suggesting that the nervous system itself can accommodate radical transformations in sensory input (18). This neuroplasticity indicates that technological mediation can become deeply integrated into the biological substrate of perception itself.

Perhaps most fundamentally, communication technologies transform the relational dimension of embodiment, altering how the body relates to other bodies and to the environment more broadly (19). As Ihde (20) argues in his phenomenology of human-technology relations, technologies are never neutral mediators but transform our bodily engagement with the world, creating new possibilities for intercorporeal connection while potentially foreclosing others.

This multidimensional transformation raises questions about the nature of bodily experience in the contemporary era. Maurice Merleau-Ponty (10) famously described the body as our “vehicle of being in the world,” emphasizing its role as the medium through which we encounter and engage with reality. Today, this vehicle has become increasingly hybrid—an assemblage of biological and technological elements that requires new phenomenological frameworks to adequately comprehend (21, 22). The challenge lies not merely in understanding individual technologies but in grasping the systematic transformation of embodied existence that emerges from their integration into the fabric of daily life.

### 2.2 Corporeality and wearable technologies: the quantified body

Wearable technologies represent perhaps the most pervasive and intimate example of how digital systems are transforming everyday bodily experience. Devices such as smartwatches, fitness trackers, biometric sensors, and other self-tracking tools have given rise to what scholars term the “Quantified Self” movement—a cultural phenomenon in which bodily experience becomes continuously measured, monitored, and analyzed through numerical data (23). Rather than constituting a universal restructuring of consciousness-body relations, this quantification process significantly influences attentional patterns and self-monitoring behaviors in ways that carry meaningful psychological and clinical implications (24, 25).

The psychological and phenomenological implications of this transformation are profound and multifaceted. Research by Ruckenstein (26) has illuminated how self-tracking creates a complex and often contradictory relationship with one’s body, where technology simultaneously serves as mirror and mediator of bodily awareness. This dual function creates what might be understood as a technological reflexivity, where the body becomes known to itself primarily through digital representation rather than direct phenomenological access. It should be noted, however, that the evidence base supports modifications in attentional patterns and self-monitoring behaviors rather than a universal restructuring of consciousness-body relations. Claims of more radical transformation require longitudinal studies specifically designed to assess changes in interoceptive processing and embodied self-knowledge attributable to wearable use, as distinct from normal behavioral adaptation.

Central to this transformation is what we might call the externalization of bodily awareness. Individuals increasingly rely on external devices for insights about their physiological states. Whether this reliance diminishes or enhances interoceptive capacity depends critically on how the external feedback is used. When individuals actively attend to their bodily sensations while simultaneously using external measures as a reference point — analogously to how dancers use mirror feedback to enhance rather than replace proprioceptive awareness — technological mediation can support and refine interoceptive sensitivity (27). Conversely, when external data are relied upon as a substitute for direct bodily attention, with individuals deliberately or habitually disengaging from their own interoceptive signals in favor of device readings, technological mediation may progressively diminish embodied self-knowledge. It is this latter pattern — reliance on external feedback accompanied by inattention to direct interoceptive experience — that characterizes the proposed construct of tracking dysperception and the ability to perceive internal bodily signals directly (28). Leder (21) described “dys-appearance” as the phenomenon by which the body erupts into conscious attention during dysfunction or pain, in contrast to its normal operational receding from awareness in everyday skilled activity. Whether continuous quantification through wearables produces an analogous disruption of pre-reflective bodily experience remains an empirical question: while some self-tracking users report heightened anxiety about bodily states and reduced confidence in unmediated bodily perception (29), the direct phenomenological parallel to dys-appearance has not been systematically tested and should be understood as a theoretical hypothesis requiring empirical validation. The implications extend beyond mere convenience to touch upon questions about the nature of embodied self-knowledge.

Parallel to this externalization, we witness a systematic fragmentation of bodily experience as the integrated totality of embodied existence becomes disaggregated into discrete, measurable parameters—steps taken, heart rate variations, sleep quality metrics, caloric expenditure. This reductive approach potentially erodes what phenomenologists understand as the holistic perception of embodied selfhood (30). The body, rather than being experienced as a unified field of sensation and possibility, becomes understood as a collection of optimizable systems, each with its own metrics and targets.

This fragmentary approach reflects what Rose (31) has identified as the emergence of “somatic individuality”—a form of selfhood where the body becomes understood primarily through isolated, measurable biomarkers rather than as an integrated experiential totality. The algorithmic nature of these technologies introduces what might be called a technological normativization of bodily experience, where “normal” or “optimal” parameters established by computational systems become authoritative references that significantly influence subjective perceptions of wellbeing (32). This creates what Canguilhem (33) would recognize as historically novel forms of “normality” and “pathology” defined not by lived experience or clinical judgment but by technological standards embedded in commercial devices.

The temporal dimension of this transformation is equally significant. The continuous monitoring enabled by wearable technologies creates what Rettberg (12) describes as a new temporality of bodily experience, where the body becomes subject to perpetual tracking and evaluation. This transforms what Husserl called the “lived time” of bodily experience—the qualitative, flowing temporality of embodied consciousness—into what Fuchs (3) might describe as an “explicit time” of continuous measurement and quantification. The rhythm of embodied life becomes synchronized with the sampling rates of digital sensors rather than the organic cycles of biological and psychological processes.

Recent longitudinal studies have documented how this quantification leads to significantly modified relationships with one’s body, relationships increasingly mediated by data and metrics rather than direct phenomenological access (24, 25). The body becomes reconceptualized as a “project” to be optimized, monitored, and continuously improved through technological intervention, with significant implications for body image formation and psychological wellbeing. This transformation of embodied existence into a technological project represents one of the most significant shifts in contemporary corporeal experience, challenging traditional phenomenological and clinical understandings of what it means to inhabit a body.

### 2.3 Virtual reality and corporeality: the simulated body

Virtual reality technologies represent a particularly significant transformation in contemporary bodily experience, enabling what might be called technologically mediated embodiment—the capacity to experience and control virtual bodies (avatars) in digitally constructed environments. These technologies create bodily experiences that challenge assumptions about the relationship between consciousness, identity, and corporeal form (34).

The mechanisms through which VR influences bodily experience operate at multiple levels of perceptual and cognitive processing. Virtual embodiment—the phenomenological illusion of possessing and controlling a virtual body—builds upon earlier research paradigms such as the “rubber hand illusion” (35) while extending these findings into fully immersive digital environments. Slater’s pioneering research (15) has demonstrated how virtual bodies can be experienced as genuinely one’s own through carefully orchestrated multisensory stimulation, suggesting that the sense of bodily ownership is far more plastic and context-dependent than previously understood.

Virtual reality does not sever embodiment from the physical body but rather temporarily remaps self-location and body ownership through multisensory stimulation. The subjective sense of “being there” in a virtual environment remains grounded in the physical body’s ongoing sensorimotor contingencies rather than representing a genuine displacement of embodied presence (15, 16). What VR produces is a context-dependent recalibration of body ownership and self-location — a phenomenon of bodily plasticity — consistent with the broader neuroscientific evidence on the malleability of the body schema (36). This virtual embodiment generates what researchers term bodily presence — the phenomenological sensation of being physically present in a virtual environment — which Ihde (20) would recognize as a genuine “embodiment relation” with technology, in which the virtual body becomes a lived extension of embodied consciousness rather than a mere representation.

Perhaps most remarkably, virtual reality enables what might be called bodily plasticity—the capacity to experience bodies with characteristics radically different from one’s physical form, including variations in gender, age, physical structure, and even species-typical features (37). This plasticity extends what Merleau-Ponty (10) described as the body’s “openness to the world”—its capacity for adaptation and extension—into previously unimaginable domains of possibility.

The transitions between virtual and physical modes of embodiment documented in the behavioral transfer literature reflect the plasticity of body schema integration rather than requiring recourse to the philosophical concept of ecstasies, which Leder (21) developed in a distinct phenomenological context not directly applicable to VR experience. The relevant empirical framework is that of body schema recalibration and sensorimotor adaptation (16, 36).

Research on virtual embodiment has revealed that the experience of inhabiting virtual bodies can significantly influence not only immediate bodily perception but also subsequent attitudes, behaviors, and cognitions in physical reality (15, 38). This phenomenon, which researchers term “behavioral transfer,” highlights the profound interconnection between virtual and physical embodied experience (39). The implications extend beyond the immediate context of VR use to suggest that technologically mediated embodiment can create lasting changes in how individuals understand and inhabit their physical bodies.

### 2.4 Augmented reality: the hybrid body

While virtual reality creates immersive technologically mediated embodiment, augmented reality (AR) technologies generate hybrid forms of embodiment by superimposing digital elements onto physical bodily experience. Technologies such as smart glasses, AR applications for smartphones, and ambient projection systems create integrated perceptual environments where digital and physical elements coexist and interact (40). This hybridization represents a distinctive modality of technological embodiment that neither fully replaces physical experience nor leaves it unmodified.

The phenomenology of augmented embodiment is characterized by several distinctive features that differentiate it from both purely physical and purely virtual experience. Perceptual simultaneity—the coexistence and integration of physical and digital elements within unified perceptual fields—creates what phenomenologists might recognize as a novel form of “intentional arc” (10) that encompasses both material and digital objects as equally present elements of the experiential world (41). This integration challenges traditional distinctions between “real” and “artificial” elements of experience by creating hybrid perceptual environments where such distinctions become phenomenologically irrelevant.

Augmented reality technologies enable what might be called sensory extension—the amplification and modification of natural perceptive capabilities through contextual digital information (42). This represents what Ihde (6) would recognize as a new form of “hermeneutic relation” with technology, where digital information becomes integrated into the interpretive frameworks through which bodily experience becomes meaningful. Rather than simply adding information to perception, AR technologies can alter the affordance structure of the environment, creating new possibilities for embodied action and understanding.

The concept of the augmented body emerges from the superimposition of digital information directly onto physical corporeal experience, creating novel forms of technologically mediated self-awareness (43). In describing this hybrid condition, Haraway (5) notion of “cyborg” existence offers a useful phenomenological metaphor for capturing the experiential blurring of organic and technological boundaries. It should be noted, however, that Haraway’s framework is primarily a political and critical analysis of technologically mediated identity, not a clinical or etiological account; its relevance to psychopathology is indirect and will be discussed in the context of social and ethical implications (Section 6**).** The augmented body is neither purely biological nor purely technological but represents a genuine hybrid that transcends these traditional categories.

Perhaps most significantly, augmented reality generates what might be called hybrid affordances—perceptions of action possibilities that integrate physical and digital elements into unified opportunities for embodied engagement (44). This transforms what Gibson (45) termed “affordances”—the action possibilities that environments offer to embodied agents—into complex hybrid structures that span physical and digital realms. These hybrid affordances suggest that the environment itself has become technologically mediated, creating new ecological niches for embodied action that did not previously exist — for instance, a surgeon navigating a complex procedure guided by real-time AR overlays of anatomical structures, or a person with visual impairment using AR-assisted navigation to orient their body through physical space in ways that would otherwise be unavailable to them. In each case, the embodied action — the specific sensorimotor engagement with the environment — is genuinely novel, made possible only through the integration of digital information into the physical perceptual field.

The psychological implications of this hybridization extend beyond immediate perceptual effects to influence aspects of embodied self-understanding. Research has demonstrated that augmented reality can modify the sense of agency and bodily ownership in ways that persist beyond the immediate technological encounter (46). These modifications potentially influence emotional regulation and decision-making processes related to bodily experience, suggesting that augmented embodiment can create meaningful changes in how individuals relate to their physical selves (47). The implications of these changes for psychological wellbeing and pathology remain an area of active investigation and clinical concern.

## 3 Emerging forms of psychopathology at the body-technology interface

The evolution of body-technology interfaces has been associated with clinically meaningful psychological presentations that challenge existing diagnostic frameworks and therapeutic approaches. These new forms of psychological distress are distinguished by their grounding in technologically mediated bodily experience, representing potentially distinct phenomena that require innovative interpretive frameworks. It should be noted that the authors cited in support of this framing address related but distinct concerns: Fuchs (48) examines the circular dynamics underlying established psychopathology rather than proposing novel disease entities arising from technology, while Lembke (49) focuses on technology-amplified existing addiction mechanisms rather than the emergence of new diagnostic categories. The conditions described in this section are therefore best understood as proposed clinical constructs warranting empirical investigation, rather than established diagnostic entities. Current evidence consists primarily of theoretical elaborations, case reports, and ethnographic observations; epidemiological data on prevalence and incidence remain limited or absent for most of the conditions described below. **Table 1** should accordingly be interpreted as a heuristic clinical framework rather than a taxonomy of validated disorders. **Table 1** provides an overview of the proposed psychopathological conditions identified in this analysis, summarizing their technological contexts, clinical presentations, underlying neurobiological mechanisms, and available or estimated prevalence data.

**Table 1:** Novel forms of body-technology psychopathology.

Disorder	Closest established framework	Technology involved	Primary symptoms	Neurobiological mechanisms	Prevalence	Key references
Filter Dysmorphia	BDD (DSM-5-TR 300.7); Body image disturbances	Digital filters, modification apps	Distress when viewing unfiltered image, social avoidance, obsessive preoccupation with digital appearance	Alterations in BDD neural circuits, abnormal visual cortex activation	Subclinical manifestations common in young adults	(50; 51; 52)
Tracking Dysperception	Illness Anxiety Disorder (DSM-5-TR 300.7); OCD spectrum	Wearable devices, fitness apps	Anxiety without tracking data, increasing difficulty trusting bodily states, dependence on external feedback	Attenuated anterior insula activation, context-dependent reduction in interoceptive attention	Increasing among habitual self-trackers	(25; 29; 53)
Virtual Depersonalization	Depersonalization/Derealization Disorder (DSM-5-TR 300.6)	VR, immersive environments	Physical body feels “less real”, difficulty readapting to physical limitations, persistent disconnection	Transient alterations in body schema processing, disrupted sensorimotor integration	Case reports, prevalence unclear	(54; 55)
Avatar-Body Identity Conflict	Gaming Disorder (ICD-11 6C51); BDD	Virtual worlds, gaming platforms	Dysphoria regarding physical body, preference for virtual embodiment, withdrawal from physical interactions	Identity processing alterations, reward circuit activation	Emerging in intensive VR users, primarily in problematic gaming contexts	(56; 57; 58)
Proprioceptive Lag Syndrome	Somatic Symptom Disorder (DSM-5-TR 300.82)	VR systems with latency	Transient delay sensation, fine-motor difficulties, compensatory strategies	Sensorimotor timing recalibration, disrupted intentional arc	Post-VR exposure transient effects documented	(59; 60, 61)
Augmented Sensorimotor Confusion	Somatic Symptom Disorder; Conversion Disorder (DSM-5-TR 300.11)	AR systems	Difficulty distinguishing physical/digital sensory feedback, disrupted affordance perception	Multisensory integration disruption	Preliminary, small observational samples	(40; 62)
Self-Tracking Addiction	OCD spectrum; Behavioral addiction (ICD-11 6C5Y)	Monitoring devices	Compulsive self-monitoring, distress when unable to track, escalating parameters	Dopaminergic reward activation consistent with behavioral addiction models	Subclinical forms increasingly common	(29; 63; 64)
Virtual Embodiment Addiction	Gaming Disorder (ICD-11 6C51); Behavioral addiction (ICD-11 6C5Y)	VR, immersive environments	Irresistible desire for virtual embodiment, tolerance, withdrawal, neglect of physical-world activities	Mesolimbic reward circuit activation, disrupted prefrontal regulation	Ethnographic evidence, prevalence unclear	(64; 65; 66)

Throughout this paper, terms such as “disorder,” “syndrome,” and “addiction” are used provisionally and heuristically to designate clinically recognizable patterns of distress warranting empirical investigation, and should not be interpreted as implying diagnostic validation. Not all engagement with body-technology interfaces carries clinical significance. The use of digital filters, self-tracking devices, and virtual reality environments is normative and, in most individuals, does not give rise to psychopathological presentations. The proposed constructs described in this section should be understood as clinically relevant only when the following features are present: symptoms persisting beyond the immediate technological context; loss of control over technology-mediated bodily behaviors; significant functional impairment in social, occupational, or interpersonal domains; active avoidance of unmediated bodily experience; marked subjective distress; compulsive engagement patterns; and inability to flexibly shift between technologically mediated and direct embodied self-representation. In the absence of such features, the behaviors described below remain within the broad spectrum of contemporary digital practice rather than constituting clinical entities.

### 3.1 Body image disorders in the digital era

Digital technologies have significantly influenced the phenomenology of body image formation, creating unprecedented mediations between embodied self-perception and external representation. Contemporary body image emerges through complex interactions between physical embodiment, digital representation, and technological mediation, generating what Cash and Smolak (67) recognize as qualitatively new forms of embodied self-relation. Featherstone (68) captures this transformation in his observation that we now inhabit a “technological imaginary” where the boundaries between physical appearance and digital representation have become increasingly permeable, creating new possibilities for both self-expression and self-alienation. Research specifically addressing technology-mediated body image has documented how exposure to digitally modified imagery and filter-based appearance modification generates distinctive patterns of body dissatisfaction and self-perception distortion that differ from those observed in pre-digital contexts (51, 52, 69).

This transformation operates through several interconnected technological mechanisms that systematically reshape how individuals perceive, evaluate, and represent their corporeal selves. The proliferation of digital filters and real-time appearance modification applications creates immediate and accessible means for altering bodily representation, while constant exposure to highly curated or algorithmically modified body images on social media platforms establishes new normative standards that may be unattainable through biological means alone. Simultaneously, the continuous evaluation of bodily parameters through self-tracking devices introduces quantified metrics into domains previously governed by subjective experience, while immersive experiences with virtual bodies that differ substantially from one’s physical form provide alternative embodied identities that may become more appealing than biological reality (69, 70).

These technological mediations have been associated with distinctive forms of body image disturbance that challenge traditional diagnostic categories and may require innovative clinical approaches tailored to the specific features of digitally mediated embodiment (71). Whether these presentations constitute genuinely novel diagnostic entities or represent technology-specific variants of established conditions such as body dysmorphic disorder remains an open empirical question. **Table 2** illustrates the differences between traditional body image disorders and their technologically mediated counterparts, highlighting how digital mediation may create clinically distinct phenomena that could require novel diagnostic and therapeutic approaches.

**Table 2:** Comparative analysis — traditional vs. Technology-mediated body image disorders.

Aspect	Traditional body dysmorphic disorder	Filter dysmorphia	Virtual embodiment disorders
Primary Focus	Actual physical features	Discrepancy between filtered/unfiltered self	Virtual vs. physical body characteristics
Distortion Source	Perceptual/cognitive dysfunction	Technological mediation and comparison	Avatar idealization and embodiment experiences
Trigger Mechanisms	Internal cognitive processes	External digital representations	Immersive virtual experiences
Avoidance Patterns	Mirrors, social situations	Unfiltered cameras, video calls	Physical world interactions
Reality Testing	Impaired regarding actual features	Impaired regarding realistic appearance standards	Confused boundaries between virtual/physical
Treatment Challenges	Cognitive restructuring, exposure	Digital literacy, technological abstinence	Virtual-physical integration therapy
Social Implications	Individual psychological distress	Cultural normalization of digital modification	Identity fragmentation, virtual preference

#### 3.1.1 Filter dysmorphia

The phenomenon of “filter dysmorphia” represents a proposed clinical construct describing distorted bodily self-perception based on comparison with digitally filtered or modified versions of oneself, creating what Chae (72) describes as a disconnection between lived embodiment and technological representation. Unlike traditional body dysmorphic disorder, which typically involves distorted perception of actual physical features, filter dysmorphia is characterized by the discrepancy between technologically mediated body image and unmodified physical reality, creating what Eco (73) might recognize as a “hyperreality” of bodily self-perception where the digital representation becomes more real than the physical referent. This construct has not yet been formally validated as a distinct diagnostic category; its delineation from body dysmorphic disorder and from normative appearance dissatisfaction in digital contexts remains an active area of investigation.

Contemporary research has documented how frequent engagement with filters and image-modifying applications leads to progressive disconnection from realistic body perception, with users gradually losing the ability to accurately assess their unmodified appearance (50, 51). Cohen et al. (74) documented significant psychological distress and behavioral avoidance in habitual users of beauty filters when confronted with unfiltered self-images, reporting patterns consistent with heightened appearance-related anxiety. These findings are suggestive of clinically meaningful psychological effects of filter use but do not constitute neurobiological evidence, and do not in themselves establish filter dysmorphia as a distinct clinical entity separate from body dysmorphic disorder or normative appearance dissatisfaction. This suggests that filter dysmorphia may share underlying neurobiological mechanisms with established body image disorders while being triggered by specifically technological stimuli. Importantly, this potential neurobiological overlap carries direct therapeutic implications: if filter dysmorphia engages neural circuits similar to those implicated in body dysmorphic disorder — including circuits involved in visual processing, appearance-related rumination, and threat appraisal — then evidence-based interventions developed for BDD, such as cognitive-behavioral therapy with exposure and response prevention (75, 76), may represent a promising starting point for addressing filter dysmorphia, adapted to account for the specific role of technological mediation in triggering and maintaining the disturbance.

The clinical presentation of filter dysmorphia includes several reported features that may differentiate it from related conditions. Individuals have been reported **to** experience significant distress when confronted with their unfiltered image, often describing feelings of shock, disappointment, or self-loathing when seeing their unmodified appearance. This distress frequently leads to avoidance of social situations where digital image control is impossible, such as video calls without filters or in-person social interactions. Many individuals develop obsessive preoccupation with how their body appears in digital images, spending excessive time adjusting filters, editing photos, or seeking the perfect digital representation. Perhaps most concerning, affected individuals demonstrate progressive difficulty recognizing the actual characteristics of their physical body, as their self-image becomes increasingly anchored to digitally modified representations. Research with adolescent and young adult social media users suggests that filter-related body image disturbance exists on a continuum rather than as a discrete diagnostic entity, with subclinical manifestations being common among individuals regularly exposed to digitally altered imagery (51, 69). This dimensional quality raises important questions about the boundaries between normative digital self-presentation practices and pathological body image distortion in contemporary culture, and underscores the need for validated assessment instruments and epidemiological studies before formal diagnostic recognition can be considered.

#### 3.1.2 Tracking dysperception

The proliferation of self-tracking devices has been associated with a distinctive form of bodily misperception characterized by excessive reliance on quantified data at the expense of subjective bodily experience. This condition, provisionally termed “tracking dysperception,” involves progressive attentional reorientation away from direct embodied self-perception toward dependence on externally measured parameters, creating what Leder (21) would describe as a technological form of bodily “absence” where direct embodied experience becomes supplanted by digital mediation (7). Importantly, this construct refers to shifts in attentional patterns and epistemic reliance on external metrics rather than to a direct impairment of interoceptive sensitivity per se; no evidence currently demonstrates that self-tracking reduces interoceptive sensitivity or produces a distinct clinical syndrome. This construct remains theoretically proposed; no validated diagnostic criteria or epidemiological prevalence data are currently available.

The relationship between self-tracking and interoceptive awareness is more nuanced than a simple unidirectional reduction. Recent evidence indicates that the direction of this relationship depends critically on the pattern and context of technology use. Haruki et al. (53) demonstrated that consistent attentional bias toward smartphone stimuli — a pattern associated with problematic use — is linked to significantly reduced interoceptive awareness, specifically in the dimensions of noticing and trusting internal bodily sensations, alongside heightened physiological reactivity consistent with behavioral addiction models. Conversely, Sumińska and Rynkiewicz (27) showed that structured real-time physiological feedback from a smartwatch, integrated within a mindfulness-based stress reduction program, enhanced mindfulness scores and supported stress reduction relative to mindfulness training alone, suggesting that wearable-mediated interoceptive feedback can facilitate rather than diminish bodily awareness when embedded in an appropriate therapeutic framework. Furthermore, Dierolf et al. (55) demonstrated that metacognitive interoceptive awareness is negatively related to susceptibility to exteroceptive manipulation of bodily self-location, indicating that individuals with stronger interoceptive awareness are more resistant to externally induced alterations of bodily self-experience. This finding suggests that interoceptive capacity may function as a moderating variable in technology-related embodiment presentations, and that the attentional reorientation characteristic of tracking dysperception — by progressively directing attention away from internal bodily signals — may increase rather than decrease vulnerability to technology-induced distortions of bodily self-experience The construct of tracking dysperception therefore refers specifically to one end of this spectrum: compulsive, unstructured self-monitoring in which external data progressively substitute for embodied self-perception, rather than representing the universal consequence of wearable device use.

Research by Ruckenstein and Schüll (25) has documented how habitual self-trackers may experience significant anxiety when unable to access their tracking data, even in situations where such monitoring has no medical necessity. This phenomenon reflects what Ihde (20) identified as the “amplification/reduction” structure of technological mediation, where certain aspects of experience become enhanced while others are systematically diminished. In the case of self-tracking, technological amplification of quantified bodily parameters occurs alongside reduction in direct interoceptive sensitivity, creating a form of technological dependence that extends beyond mere convenience to touch upon questions of embodied self-knowledge.

The proposed symptom profile of tracking dysperception includes several interconnected features that collectively undermine natural bodily awareness. Affected individuals demonstrate increasing difficulty perceiving and trusting bodily states without external device confirmation, often expressing doubt or anxiety about their physical condition when tracking data is unavailable. This dependency generates significant anxiety when monitoring systems are inaccessible, sometimes leading to panic or distress that appears disproportionate to actual health risks. Perhaps most problematically, individuals begin to devalue subjective bodily experience in favor of “objective” technological data, dismissing their own sensations when they conflict with device readings. This epistemic privileging of technological measurement over embodied knowledge often leads to compulsive self-monitoring behaviors and diminished capacity to interpret bodily signals in the absence of quantified metrics (24).

Esmonde (29) ethnographic work with fitness tracker users provides observational documentation of how technological mediation can override embodied self-perception, with individuals dismissing clear bodily signals such as fatigue, pain, or hunger when they contradicted device readings. These ethnographic observations offer valuable qualitative evidence but do not constitute epidemiological or clinical data sufficient to establish tracking dysperception as a distinct disorder. Prospective clinical studies with standardized assessment instruments are needed to determine prevalence, severity thresholds, and differentiation from existing anxiety and obsessive-compulsive spectrum conditions (30).

### 3.2 Embodiment disorders in virtual environments

The immersive nature of virtual reality technologies has been associated with distinctive psychological presentations that reflect the challenges posed by inhabiting simulated bodies in digital environments. While research supports that VR can alter embodiment and self-perception, the effects documented in the literature are typically transient; there is currently no empirical evidence that VR use gives rise to distinctive, lasting disorders that challenge established clinical understandings of identity or corporeal form. These conditions arise from the complex interactions between consciousness, virtual embodiment, and the subsequent return to physical reality (16, 77). Madary and Metzinger (78) are cited here solely for their identification of potential psychological risks associated with immersive VR warranting precautionary attention, not as documentation of novel diagnostic categories. The proposed constructs described below are grounded in documented neurobiological and perceptual phenomena but have not yet been validated as independent diagnostic categories and require systematic clinical investigation before any diagnostic status can be considered.

#### 3.2.1 Virtual depersonalization disorder

Virtual depersonalization disorder is a proposed construct describing persistent feelings of estrangement from one’s physical body following prolonged or intense experiences of virtual embodiment. Unlike traditional depersonalization disorder, which typically involves generalized feelings of unreality or detachment, the virtual variant is theorized to be specifically characterized by its connection to avatar embodiment experiences, creating what Merleau-Ponty (10) might describe as a technologically induced disturbance in the “body schema” that persists beyond the immediate virtual experience (54).

The phenomenology of this proposed condition includes several reported features that may differentiate it from related dissociative disorders. Individuals have reported that their physical body feels less “real” or “present” compared to the virtual body they inhabited, sometimes describing their flesh-and-blood form as foreign or inadequate. This is frequently accompanied by difficulty readapting to the limitations of physical embodiment after experiencing the enhanced capabilities or altered characteristics available through virtual bodies. Many individuals reporting such experiences perceive the physical world as “less vivid” or “less engaging” compared to virtual environments, leading to persistent desire to return to virtual embodied experiences. Perhaps most concerning, some individuals report persistent sensations of disconnection between motor intentions and actual physical movements, as if their consciousness remains partially anchored to virtual rather than physical embodiment.

Aardema et al. (54) documented transient dissociative effects following VR exposure, including short-term reductions in sense of presence in objective reality. These findings provide a plausible phenomenological basis for the proposed construct but do not demonstrate neurobiological or sensorimotor alterations persisting for hours or days, nor do they establish virtual depersonalization as a condition distinct from known depersonalization/derealization presentations. The work of Yee and Bailenson (39) on the “Proteus Effect” suggests that virtual embodiment can produce behavioral modifications that persist after leaving virtual environments, further supporting the theoretical plausibility of the construct without constituting clinical validation.

Relevant to understanding individual variability in susceptibility to these presentations, Dierolf et al. (55) demonstrated that metacognitive interoceptive awareness is negatively related to the exteroceptive malleability of bodily self-location: individuals with stronger interoceptive awareness are less susceptible to experimentally induced alterations of bodily self-experience. This suggests that interoceptive capacity may moderate vulnerability to VR-induced embodiment disturbances, a hypothesis that warrants direct empirical investigation in clinical populations.

Case studies document individuals experiencing depersonalization symptoms following intensive VR gaming sessions, with symptoms sometimes persisting beyond the immediate exposure period. These observations are clinically suggestive but remain at the level of anecdotal and case report evidence; systematic case series and controlled clinical studies are needed to characterize the condition, establish diagnostic boundaries, distinguish it from pre-existing depersonalization/derealization disorder, and determine whether prevalence reaches clinically meaningful levels (78).

#### 3.2.2 Avatar-body identity conflict

Extended engagement with virtual environments has been observed to generate profound identity conflicts between virtual self-representation and physical embodiment, particularly when avatars possess characteristics that differ significantly from the user’s biological body. This phenomenon, which Yee (57) has termed avatar-body identity conflict, occurs when individuals develop strong psychological attachment to virtual bodily identities that conflict with their physical embodiment, creating what Turkle (79) might describe as a technologically mediated “second self” that competes with rather than complements physical identity. As with other conditions described in this section, this construct is theoretically elaborated and supported by qualitative evidence but awaits formal clinical validation.

It is important to note that research on avatar embodiment generally shows transient and reversible effects on body perception. Persistent dysphoria or enduring bodily alienation attributable to avatar use has not been clearly documented in general populations. Identity-related distress arises primarily in specific contexts such as problematic gaming or pre-existing psychological vulnerability, rather than as an inherent consequence of virtual embodiment (57, 58).

The reported clinical manifestations of avatar-body identity conflict encompass both psychological and behavioral dimensions that can significantly impact daily functioning. Individuals may experience profound dysphoria regarding their physical body after extended identification with idealized or substantially different avatar bodies, sometimes describing their biological form as disappointing, limiting, or alien. This often generates cognitive dissonance between virtual and physical self-image, with individuals feeling more authentic or comfortable in virtual form than in physical reality. Some affected individuals make attempts to modify their physical body to align more closely with avatar characteristics, ranging from cosmetic changes to more substantial surgical interventions. Social withdrawal from physical-world interactions often accompanies preference for virtual social engagement, where the individual can maintain their preferred embodied identity. Perhaps most diagnostically significant, individuals experience distress when unable to access their preferred virtual bodily experience, sometimes displaying withdrawal-like symptoms when separated from virtual environments.

Research by Bessière et al. (56) has documented how many individuals create avatars representing idealized versions of themselves, which can lead to increasing discrepancy between virtual and physical self-perception over time. Taylor and Schroeder (80) ethnographic research with online world participants provides documentation of the emotional significance that virtual embodiment can acquire, including cases where individuals experienced profound grief when losing access to long-term avatars. These observations, while clinically evocative, derive primarily from ethnographic and qualitative sources; prospective clinical studies are required to establish whether these presentations constitute a distinct clinical syndrome, determine prevalence, characterize severity, and differentiate from established conditions such as body dysmorphic disorder, depersonalization disorder, and gaming disorder as defined in ICD-11 and DSM-5-TR (57, 58).

### 3.3 Disorders of technologically mediated proprioception

Technologies that mediate bodily experience can interfere with proprioceptive mechanisms, generating clinically relevant presentations that challenge established understandings of sensorimotor integration and embodied cognition. These conditions arise when technological interfaces disrupt the normally seamless coordination between motor intention, sensory feedback, and proprioceptive awareness that characterizes healthy embodied experience (22).

#### 3.3.1 Proprioceptive lag syndrome

Proprioceptive lag syndrome is a proposed construct describing persistent discrepancy between bodily movements and sensory feedback, phenomenologically similar to the experience of “lag” or delay in virtual reality systems but persisting even in non-mediated environments. Individuals have reported sensations of delay or misalignment between motor intention and movement experience, representing what Merleau-Ponty (10) would describe as a disruption in the “intentional arc” that normally connects perception and action in seamless embodied engagement with the world (60, 61).

Research by Fulvio et al. (59) has demonstrated that exposure to VR systems with significant latency can temporarily recalibrate sensorimotor timing mechanisms, with effects persisting after leaving the virtual environment. This constitutes empirical evidence for a transient post-VR sensorimotor aftereffect; whether such effects reach clinical significance, persist sufficiently to constitute a syndrome, and occur at meaningful prevalence in real-world VR use contexts remains to be established through systematic clinical observation (81).

#### 3.3.2 Augmented sensorimotor confusion disorder

The integration of digital information with physical sensory experience in augmented reality environments has been associated with forms of sensorimotor confusion in which the integration between physical sensory signals and digital information becomes problematic. This proposed disorder manifests as difficulty in distinguishing between physical and digital sensory feedback, creating what Gibson (45) might recognize as a disruption in the perception of environmental “affordances” due to the hybrid nature of the augmented perceptual field (40).

These findings are preliminary and based on small observational samples; controlled clinical studies are needed before this presentation can be characterized as a distinct disorder (62).

### 3.4 Behavioral addictions related to technologically mediated bodily experience

Body-technology interfaces may generate specific forms of behavioral addiction centered on technologically mediated bodily experience, challenging traditional addiction models that focus primarily on substance use or gambling behaviors. Lembke (49) has documented how existing addiction mechanisms — particularly dopaminergic reward circuits — are amplified by technological design features; the conditions described below should be understood within this framework of technology-amplified addiction processes rather than as categorically novel addiction entitie**s.** These conditions reflect the capacity of body-technology interfaces to activate reward systems and create compulsive behavioral patterns that interfere with daily functioning and psychological wellbeing (64, 82).

#### 3.4.1 Self-tracking addiction

Self-tracking addiction is a proposed construct describing compulsive self-monitoring through tracking devices, with significant distress when unable to collect bodily data and progressive escalation in the quantity and types of parameters monitored. Schüll (13) observes that the continuous feedback loops created by self-tracking technologies can generate powerful reinforcement mechanisms similar to those observed in other behavioral addictions. Whether this constitutes a distinct addiction entity or represents a technology-specific manifestation of obsessive-compulsive or anxiety spectrum pathology remains to be determined through controlled clinical investigation.

The proposed diagnostic criteria encompass both behavioral and psychological dimensions that collectively create significant functional impairment. Individuals display persistent preoccupation with monitoring bodily parameters, often thinking about tracking data even when not actively engaged with devices. This is accompanied by significant distress when unable to perform tracking, sometimes leading to panic or anxiety when devices malfunction or data is unavailable. The condition typically involves escalation in the frequency and complexity of monitoring, with individuals progressively adding new parameters or increasing sampling frequency in ways that begin to dominate daily life. Despite recognizing negative consequences, affected individuals continue the behavior and make repeated failed attempts to reduce or control their tracking behaviors (63).

Research by Esmonde (29) has documented withdrawal-like symptoms when individuals are separated from their tracking devices, including anxiety, irritability, and compulsive checking behaviors. These observations are consistent with behavioral addiction models (64) and warrant systematic clinical investigation, including comparison with existing ICD-11 and DSM-5-TR criteria for other behavioral addictions to determine whether a distinct category is justified.

Notably, the interoceptive dysregulation documented in individuals with problematic technology engagement — specifically, reduced capacity to notice and trust internal bodily signals (53) — may represent a shared neuropsychological substrate linking self-tracking addiction to other behavioral addiction presentations, and warrants investigation as a potential diagnostic marker.

#### 3.4.2 Virtual embodiment addiction

Virtual embodiment addiction is a proposed construct describing compulsive pursuit of embodiment experiences in virtual bodies, often with marked preference for bodies possessing characteristics unavailable in physical reality. Rather than invoking Haraway (5) political framework — which addresses identity and power rather than addiction mechanisms — this condition is better understood through established behavioral addiction models (64, 82) and the documented capacity of idealized avatar embodiment to activate mesolimbic reward circuits (39).

The proposed symptom profile includes several features that parallel established addiction criteria while reflecting the unique aspects of virtual embodiment. Individuals experience irresistible desire to access virtual embodiment experiences, often organizing their lives around opportunities for virtual engagement. The condition typically involves tolerance, with progressive need to increase the duration or intensity of virtual embodiment to achieve the same psychological effects. Withdrawal symptoms manifest as irritability, anxiety, or dysphoria when unable to access preferred virtual bodies. Perhaps most concerning, individuals often abandon significant activities in the physical world, neglecting work, relationships, or health needs to maintain virtual presence (65).

Ethnographic studies by King and Warren (83) have documented cases where individuals prioritize virtual embodiment experiences over basic physical health needs. Research by Gackenbach and Bown (66) provides evidence for the profound psychological integration that virtual embodiment can achieve, with some intensive users reporting dreams that incorporate their avatar bodies. These observations are clinically suggestive but derive from ethnographic rather than clinical sources. Systematic case series and controlled studies comparing virtual embodiment addiction presentations with established gaming disorder criteria (ICD-11) are needed to determine whether a distinct diagnostic construct is warranted (84).

## 4 Neurobiological foundations and clinical approaches to technology-mediated body alterations

The psychological presentations associated with body-technology interfaces described in Section 3 are plausibly grounded in neurobiological mechanisms that have been documented in experimental research. It is important to note, however, that the evidence base supports transient neurophysiological and experiential effects rather than lasting neural reorganization from prolonged technology use; the two should not be conflated. Understanding the relevant mechanisms — within their documented scope — is nonetheless essential for developing theoretically informed therapeutic approaches (36).

### 4.1 Plasticity of neural body representation

The human brain maintains neural representations of the body that are remarkably plastic and can be modified through experience. This plasticity forms the foundation for the ability to incorporate tools and technologies into the bodily self (8). As Merleau-Ponty (10) observed, the body schema demonstrates a fundamental openness to technological incorporation, a principle now supported by extensive neuroscientific research.

The evidence base for technology-induced neural plasticity must be interpreted carefully with respect to the duration and reversibility of documented effects. Neuroimaging and neurophysiological studies have documented how technology use can modify neural activity and body representations, though the majority of documented effects are transient rather than representing lasting structural reorganization. Cardinali et al. (9) found that tool use can induce changes in body metric representations affecting subsequent movements performed without the tool, demonstrating genuine incorporation of tools into the body schema at a neural level. Blanke and Metzinger (2009), frequently cited in this context, explicitly describe temporary VR-mediated effects on bodily self-consciousness rather than lasting neural reorganization, and should be interpreted accordingly.

These modifications involve multiple neural systems:

Somatotopic maps in sensorimotor cortex: Research by Kannape et al. (85) has shown that virtual body ownership can induce measurable changes in motor cortical excitability, representing transient modulation of body representations rather than lasting reorganization.

Multisensory integration in the parietal lobe: Studies have revealed how virtual embodiment modulates activity in parietal regions responsible for integrating visual, proprioceptive, and tactile information into a coherent body representation (86). These modulations are documented during and immediately following virtual embodiment experiences.

Body image processing in the temporo-parietal junction: Neuroimaging research by Limanowski and Blankenburg (87) has identified this region as crucial for maintaining the sense of body ownership, with activity patterns altered during virtual embodiment experiences.

Agency and body ownership circuits: Research has mapped the neural networks involved in the sense of agency and ownership and their response to multisensory manipulation (36, 88).

Contemporary body-technology interfaces exploit these plasticity mechanisms through transient multisensory manipulation. Whether prolonged or repeated exposure produces lasting modification of neural body representations beyond these well-documented transient effects remains an open empirical question that the available evidence does not currently resolve.

### 4.2 Interoception and technological mediation

Interoception, the perception of the body’s internal states, is a fundamental process for emotional regulation and self-awareness (89). Damasio (90) influential work on somatic markers established how interoceptive signals provide essential information for decision-making and emotional processing (91). Recent studies suggest that technological mediation of bodily experience may influence interoceptive processes, though the nature and persistence of these effects depends critically on the pattern of technology use.

Farb et al. (92) demonstrated that attention to external metrics about bodily states can attenuate activation in the anterior insula, a key region for interoceptive awareness. This finding suggests a potential neural mechanism for what Leder (21) called the “absent body” — a state where direct bodily awareness is supplanted by abstract, technologically mediated representations. This effect reflects attentional redirection rather than structural impairment of interoceptive capacity.

Several mechanisms have been proposed through which technology use may influence interoceptive processing:

Attentional redirection toward quantified parameters can modify the salience of internal bodily signals: Using functional MRI, Mehling et al. (28) documented how focus on numerical representations of bodily states redirects attentional resources away from direct interoceptive processing pathways. This attentional effect is consistent with the construct of tracking dysperception described in Section 3, with the important qualification that it reflects shifts in attentional allocation rather than impairment of interoceptive sensitivity per se.

Problematic technology engagement is associated with reduced interoceptive awareness: Haruki et al. (53) demonstrated that consistent attentional bias toward smartphone stimuli — associated with problematic use patterns — is linked to significantly reduced scores on the noticing and trusting subscales of the MAIA, alongside heightened physiological reactivity. This finding extends the interoception-technology relationship beyond laboratory attention paradigms to naturalistic patterns of technology engagement.

Insula processing can be modulated by virtualized bodily experiences: Research by Suzuki et al. (46) using cardio-visual feedback in virtual environments demonstrated significant modulation of insular activity during technologically mediated bodily experiences. These effects were documented during the virtual experience itself.

Conversely, structured use of wearable physiological feedback within mindfulness-based frameworks can enhance interoceptive awareness rather than diminish it (27), underscoring that the direction of technology’s effect on interoception is context- and pattern-dependent rather than uniformly negative.

Relevant to understanding individual vulnerability, Dierolf et al. (55) demonstrated that metacognitive interoceptive awareness is negatively related to susceptibility to exteroceptive manipulation of bodily self-location. This suggests that interoceptive capacity may function as a protective factor moderating vulnerability to technology-induced distortions of bodily self-experience, with implications for identifying at-risk individuals and developing targeted interventions. As Tsakiris (93) argues, the predictive processing framework suggests that excessive reliance on external body models may alter the hierarchical processing of bodily signals, potentially undermining the primacy of direct interoceptive experience.

### 4.3 Reward circuits and behavioral addictions related to technology

The behavioral presentations associated with technologically mediated bodily experience involve reward circuits, particularly the dopaminergic pathways implicated in other addictive behaviors (94). It is important to note that the evidence base for dopaminergic involvement in technology-specific presentations derives primarily from research on substance addiction and general addiction neurobiology rather than from studies directly examining technology-mediated bodily experience; direct extrapolation should therefore be made with caution.

As Griffiths (64) has argued within a behavioral addiction framework, technologically mediated experiences may activate neural mechanisms overlapping with those underlying other addictive behaviors, despite their apparent differences. Several neural systems are relevant to this discussion:

The mesolimbic dopaminergic system is activated by rewarding experiences and plays a central role in reinforcement learning and addictive behavior (94). Whether immersive virtual reality experiences involving idealized avatar embodiment produce dopamine release in the ventral striatum comparable to that documented in substance addiction remains to be demonstrated through direct neuroimaging studies specifically targeting these experiences.

The nucleus accumbens is involved in reward-seeking behaviors. Functional MRI research has shown that positive feedback activates reward circuits consistent with behavioral addiction models (95), though studies specifically examining self-tracking feedback in this context are limited.

The prefrontal cortex is implicated in the regulation of compulsive behaviors. Research on neural correlates of self-control in technology use has identified disrupted connectivity between prefrontal regulatory regions and limbic reward areas in individuals showing problematic patterns (96). This finding is consistent with, though not specific to, the behavioral addiction presentations proposed in Section 3.

The framework of technology-amplified addiction — in which existing dopaminergic reward mechanisms are engaged and potentially intensified by specific features of technological design, such as variable reward schedules and optimized feedback — provides a more empirically defensible account than claims of categorically novel neurobiological pathways (49, 64). Targeted neuroimaging studies specifically examining self-tracking and virtual embodiment contexts are needed to move beyond this theoretical framework toward direct empirical characterization of the relevant mechanisms.

## 5 Clinical approaches to technology-related bodily distress

The psychological presentations associated with body-technology interfaces described in the preceding sections — while not yet constituting validated diagnostic entities — raise genuine clinical questions about assessment and intervention. The approaches described in this section should be understood as clinically informed responses to technology-amplified presentations of established conditions, and as frameworks for addressing the specific features of technologically mediated distress within existing therapeutic paradigms, rather than as treatments for novel and independently validated disorders. The challenge for contemporary clinicians lies in developing approaches that acknowledge the unique characteristics of technologically mediated experience while remaining grounded in the evidence base for established conditions (48, 97).

### 5.1 Clinical assessment of technology-related bodily experience

The clinical assessment of technology-related psychological presentations requires attention to dimensions of experience that conventional assessment tools — developed within frameworks that assume unmediated embodiment — may not adequately capture. This does not imply that specialized instruments for novel disorders are needed, but rather that existing validated tools should be applied with sensitivity to the specific ways in which digital technologies influence bodily self-relation and embodied identity (82).

Krueger and Osler (98) discuss the phenomenological and engineering dimensions of technologically mediated experience; their work is referenced here in the more limited sense of informing clinical sensitivity to how technologies become meaningful within particular embodied subjectivities, rather than as a basis for specialized diagnostic instruments.

Several assessment domains warrant attention in individuals presenting with technology-related distress. The assessment of dependence on technologically mediated bodily experiences requires attention to contextual factors and compensatory motivations rather than relying solely on behavioral symptom counts. Research by Kardefelt-Winther (82) suggests that problematic technology engagement often reflects complex interactions between individual vulnerabilities, technological affordances, and broader contextual factors, which effective clinical assessment must capture. Evaluation of dissonance between physical and virtual or augmented bodily experience may be relevant for individuals presenting following intensive VR use or prolonged engagement with appearance-modifying technologies. Phenomenologically informed assessment approaches, building on the foundational work of Fuchs and Schlimme (1) on embodiment and psychopathology, can help access the lived experience of this dissonance within existing clinical interview frameworks. Fuchs’ work addresses the circular dynamics of embodied experience and psychopathology broadly and provides a theoretically informed lens for clinical inquiry rather than a framework specifically developed for technology-induced disorders.

Assessment of interoceptive awareness using validated instruments — particularly the Multidimensional Assessment of Interoceptive Awareness (MAIA-2; 28) — can measure shifts in bodily self-knowledge relevant to technology-related presentations such as tracking dysperception (99). The MAIA-2 is a validated psychometric instrument with established psychometric properties and is recommended here for its documented relevance to interoceptive processing rather than as a technology-specific tool.

Regarding the assessment instruments described in earlier versions of this manuscript: The Self-Tracking Dependency Scale (STDS), Technological Body Dissonance Inventory (TBDI), and Semi-structured Interview for Digital Bodily Experience (SIDBE) are conceptual proposals rather than validated clinical instruments. No published literature documents their development, psychometric properties, validity, or clinical use. They are presented here as directions for future instrument development rather than as established tools, and clinicians should rely on validated existing instruments — including the MAIA-2, established body image scales, and validated addiction measures — pending formal development and validation of technology-specific assessment tools. The Virtual Embodiment Questionnaire (VEQ; 100) has been developed within the context of VR research and may offer useful dimensions for clinical inquiry regarding virtual embodiment experiences, though its clinical validation remains limited. **Table 3** should be interpreted as a heuristic framework for assessment domains rather than as a battery of validated instruments.

**Table 3:** Integrated assessment framework for technology-mediated embodiment disorders.

Assessment domain	Instruments	Key measures	Clinical applications	Key references
Virtual Embodiment Identification	Virtual Embodiment Questionnaire (VEQ)	Presence, agency, identity transfer, body ownership in VR	Assess avatar-body conflicts, virtual depersonalization	(100)
Self-Tracking Dependency	Self-Tracking Dependency Scale (STDS) — *proposed instrument*	Compulsive monitoring, withdrawal symptoms, functional impairment	Evaluate self-tracking addiction, tracking dysperception	(25; 63)
Technological Body Dissonance	Technological Body Dissonance Inventory (TBDI) — *proposed instrument*	Discrepancy experience, mediated vs. direct embodiment	Measure filter dysmorphia, AR/VR integration issues	(1)
Digital Bodily Experience	Semi-structured Interview for Digital Bodily Experience (SIDBE) — *proposed instrument*	Phenomenological exploration, narrative integration, meaning-making	Comprehensive qualitative assessment, treatment planning	(1; 101)
Interoceptive Sensitivity	Multidimensional Assessment of Interoceptive Awareness (MAIA-2)	Body awareness, attention regulation, emotional awareness	Assess impact of technological mediation on bodily awareness	(28; 53)
Technology-Mediated Body Image	Modified Body Image Assessment	Digital vs. physical self-perception, filter dependency, unrealistic standards	Evaluate filter dysmorphia severity and progression	(51; 52)

STDS, TBDI, and SIDBE are proposed conceptual instruments pending formal psychometric development and validation. Clinicians should currently rely on validated tools — including the MAIA-2, established body image scales, and validated addiction measures — pending formal development of technology-specific instruments.

### 5.2 Therapeutic approaches

The therapeutic approaches described below are grounded in established evidence bases for recognized conditions — including body dysmorphic disorder, obsessive-compulsive disorder, gaming disorder, and anxiety disorders — and their potential applicability to technology-amplified presentations of these conditions. The framing is not that novel disorders require specially designed treatments, but rather that existing evidence-based approaches may require specific adaptation to address the technological features of presentation that are increasingly encountered in clinical practice (**Table 4**).

**Table 4:** Therapeutic approaches matrix for technology-related embodiment disorders.

Therapeutic approach	Target disorders	Key interventions	Mechanisms of action	Evidence level	Key references
Somatic Mindfulness	Tracking Dysperception, Virtual Depersonalization	Body awareness cultivation, interoceptive focusing, breathing practices	Strengthen pre-reflective bodily awareness, reduce technological dependence	Emerging evidence	(102; 103; 104)
Modified CBT	Filter Dysmorphia, Self-Tracking Addiction	Cognitive restructuring for digital standards, graduated exposure to unfiltered images	Challenge unrealistic technological standards, build tolerance for authentic appearance	Adapted protocols showing promise	(75; 109; 110)
Sensorimotor Therapy	Virtual Depersonalization, Proprioceptive Disorders	Bottom-up processing, embodied present-moment awareness, grounding techniques	Restore physical embodied presence, integrate sensorimotor experience	Clinical case reports	(105; 106; 108)
Therapeutic VR	Filter Dysmorphia, Avatar-Body Conflicts	Controlled virtual embodiment, corrective avatar experiences, body acceptance training	Leverage VR for positive body image experiences, realistic self-perception	Pilot studies	(112; 113)
Phenomenological Therapy	All technology-mediated disorders	Existential exploration, meaning-making of technological embodiment, authenticity focus	Understand significance within life narrative, develop conscious technology relationships	Theoretical framework	(1; 101; 118)
Digital Detox Programs	Self-Tracking Addiction, Virtual Embodiment Addiction	Structured technology abstinence, alternative embodied activities, relapse prevention	Break compulsive patterns, restore natural bodily awareness	Behavioral addiction models adapted	(64; 82; 111)
Augmented Biofeedback	Tracking Dysperception	Technology-enhanced interoception, bridging digital-to-direct awareness	Use technology to restore rather than replace embodied awareness	Emerging evidence	(27; 92)

#### 5.2.1 Approaches oriented toward bodily awareness

Therapeutic approaches focused on enhancing bodily awareness may be valuable for individuals presenting with technology-related body image disturbance, compulsive self-monitoring, or dissociative-like presentations following intensive VR use. These approaches aim to support reconnection with direct embodied experience where technological mediation has come to substitute for it (102).

Mindfulness-based interventions, building on the foundational work of Kabat-Zinn (103), focus on cultivating direct awareness of bodily sensations and proprioceptive feedback. Kabat-Zinn’s MBSR was developed for stress reduction and chronic pain and has not been specifically validated for technology-related embodiment presentations. However, the general evidence base for mindfulness-based interventions in body image disturbance and anxiety (104) supports their potential applicability to technology-amplified presentations of these conditions, pending specific validation studies.

Sensorimotor therapy approaches, developed by Ogden and colleagues, address dysregulation of embodied experience through bottom-up processing of sensorimotor awareness (105, 106). This is a trauma-focused intervention with an established evidence base for trauma and attachment-related presentations. Its application to technology-related embodiment presentations is a theoretical extension that has not yet been empirically validated in this specific context, though its focus on restoring embodied presence may be relevant for presentations involving disconnection from physical bodily experience.

Experiential focusing, developed by Gendlin (107), provides methods for accessing the bodily “felt sense” that exists beyond technologically mediated representations, helping individuals develop capacity to recognize and work with subtle bodily sensations carrying emotional meaning.

Bodily grounding techniques, integrated with trauma-informed approaches by van der Kolk (108), may be relevant for individuals experiencing dissociative-like presentations following intensive virtual embodiment. These techniques draw on an established clinical literature for dissociative presentations and are proposed here as potentially applicable to technology-related dissociative phenomena, not as validated treatments for a novel disorder.

#### 5.2.2 Adapted cognitive-behavioral interventions

Cognitive-behavioral approaches represent the most empirically grounded option for technology-related presentations, given their established evidence base for the conditions most relevant to this context. The key clinical reframing is that CBT addresses technology-amplified variants of established conditions — body dysmorphic disorder, OCD-spectrum compulsive monitoring, gaming disorder — rather than novel diagnostic categories requiring entirely new therapeutic frameworks.

Cognitive restructuring interventions, building on the established evidence base for body image disturbance (109), can be applied to dysfunctional beliefs related to digitally idealized bodies and filter-mediated self-perception. McLean et al. (110) have demonstrated how cognitive techniques can address distortions arising from exposure to digitally modified bodies, supporting critical awareness of how technological mediation shapes body image.

Graduated exposure interventions, adapted from protocols for body dysmorphic disorder (75), offer systematic approaches for presentations involving avoidance of unmediated bodily experience, such as filter dysmorphia. These interventions gradually reintroduce individuals to unmodified bodily perception through structured exposure within established BDD treatment frameworks.

Behavioral management techniques, drawing on established interventions for gaming disorder (111), can address compulsive self-monitoring and other problematic patterns of technological engagement. King et al. (111) specifically addresses gaming disorder as an established diagnostic entity; these techniques are applicable to technology-related compulsive behaviors within the framework of existing behavioral addiction treatment rather than as treatments for independently validated novel disorders.

Response prevention interventions, adapted from OCD protocols (76), have particular relevance for repetitive checking and reassurance-seeking behaviors in technology-mediated body presentations, addressing the compulsive patterns that maintain technology-related anxiety within an established clinical framework.

#### 5.2.3 Technologically mediated therapeutic approaches

Several technology-based therapeutic modalities have an emerging evidence base that may be applicable to technology-related presentations.

Modified virtual reality exposure therapy (VRET) protocols, building on research by Riva and Melis (112) for body image disturbances, can provide graduated exposure to unmodified body images within controlled therapeutic contexts. This application has an emerging evidence base within body image research and represents a grounded extension of established VRET methodology.

Augmented biofeedback interventions may help reestablish connection with natural interoceptive processes by using technology to enhance rather than replace direct bodily awareness (92). The goal is to use technology as a bridge back to direct interoceptive awareness, consistent with the evidence from Sumińska and Rynkiewicz (27) showing that structured wearable feedback within a mindfulness framework can enhance interoceptive sensitivity.

Therapeutic virtual embodiment experiences, based on research by Serino et al. (113), demonstrate how carefully designed avatar experiences can positively influence body perception and acceptance, with an emerging evidence base in body image intervention research.

Bodily mindfulness applications use tracking data to enhance rather than replace natural bodily awareness. Sumińska and Rynkiewicz (27) demonstrated that supplementing mindfulness-based stress reduction with real-time physiological feedback from a smartwatch enhanced mindfulness scores and supported stress reduction relative to mindfulness training alone, suggesting that wearable-mediated physiological feedback can support rather than undermine interoceptive awareness when integrated within an appropriate contemplative framework. Beyond body image applications, virtual reality technology offers promising avenues for delivering evidence-based psychosocial interventions for already-recognized psychiatric conditions, potentially enhancing both efficacy and user engagement. In the context of schizophrenia spectrum disorders, several evidence-based psychosocial interventions — including cognitive remediation, metacognitive training, social skills training, psychoeducation, and cognitive behavioral therapy — have been established as effective treatments, with future research increasingly exploring the opportunities provided by novel technologies in their delivery (114). Cognitive remediation in particular appears well-suited to VR-based enhancement: immersive, ecologically valid virtual environments may increase treatment engagement and facilitate the transfer of cognitive gains to real-world functioning, with preliminary evidence suggesting improvements in attention, memory, and executive functioning alongside high levels of user acceptability (115, 116). These developments suggest that the same technological interfaces that may contribute to psychopathological presentations in some contexts can, when carefully designed and clinically implemented, serve as powerful vehicles for delivering established treatments with enhanced accessibility, engagement, and ecological validity — a paradox that underscores the dual potential of body-technology interfaces as both sources of risk and instruments of healing.

#### 5.2.4 Phenomenological and existential approaches

Phenomenological and existential therapeutic approaches offer meaningful frameworks for addressing questions of authenticity, meaning, and identity in technology-mediated lives. These approaches are presented here as clinically valuable for addressing existential dimensions of technology-related distress — questions about authentic living, meaning, and self-understanding in a digitally mediated world — rather than as treatments for validated technology-specific disorders.

Existential therapeutic frameworks (117) can help individuals explore the significance of technological engagement for authentic existence, addressing how technological mediation relates to themes of freedom, responsibility, and meaning.

Phenomenological analysis building on Heidegger (118) distinctions between ready-to-hand and present-at-hand modes of engagement can help individuals recognize how technology transforms their mode of embodied existence and develop more conscious relationships with technological tools. Heidegger’s framework is introduced here as a conceptual lens for understanding the phenomenology of technological engagement rather than as direct clinical evidence for the existence of technology-induced pathology.

Hermeneutic approaches inspired by Ricoeur (101) work on narrative identity can support individuals in integrating their technological embodiment experiences within broader life narratives and developing more coherent self-understanding.

Intercorporeal analysis building on Merleau-Ponty (10) understanding of intercorporeality can help individuals explore how technological mediation transforms embodied relating to others, addressing disruptions in interpersonal connection that may accompany technology-related distress. These philosophical frameworks provide a theoretically rich vocabulary for clinical inquiry but are not in themselves evidence of clinical pathology; their value lies in orienting therapeutic attention toward dimensions of lived experience that more behavioral approaches may not adequately address.

## 6 Ethical and social issues

The proliferation of body-technology interfaces raises ethical and social questions that extend beyond individual clinical concerns to encompass broader societal implications. These issues are addressed here as important ethical and social concerns in their own right, independently of whether the psychological presentations described in earlier sections constitute validated diagnostic entities. The ethical problems of unequal access, exploitative design, and structural power asymmetries are significant regardless of the nosological status of technology-related psychological distress. These issues require careful consideration of how technological mediation of embodied experience intersects with questions of justice, autonomy, and human dignity (6, 119). The ethical implications of body-technology interfaces operate at two distinct levels. At the individual clinical level, ethics concerns the responsibility to assess technology use contextually, avoid pathologizing normative digital practices, and support flexible rather than compulsive engagement. At the broader sociotechnical level, critical attention must be directed toward the algorithmic normalization of idealized body standards embedded in commercial platforms, the commercial interests of self-tracking industries that may incentivize compulsive monitoring, differential access to body-modifying technologies across socioeconomic and demographic lines, and the largely unregulated responsibility of technology designers whose choices shape the conditions within which embodied distress emerges.

### 6.1 Accessibility and inequalities in technological bodily experience

The distribution of access to technologies that mediate bodily experience reflects and potentially amplifies existing social inequalities, creating differential opportunities for both enhancement and vulnerability. As Haraway (5) presciently observed in her foundational work on cyborg identity, technological embodiment never occurs in a social vacuum but is always embedded within existing power structures that may perpetuate or transform social hierarchies. The unequal distribution of access to body-technology interfaces creates multiple forms of stratification with direct implications for clinical practice and public health, independently of claims about novel diagnostic categories (120).

Access to advanced enhancement technologies creates new forms of embodied privilege, where individuals with greater economic resources can access sophisticated tools for bodily optimization, augmentation, and therapeutic intervention. This differential access may exacerbate existing health disparities while creating entirely new categories of advantage and disadvantage based on technological embodiment capabilities. Research by Schüll (13) has documented how digital health technologies often reproduce existing healthcare disparities, with implications that extend beyond individual treatment access to encompass questions about what constitutes normal or optimal embodied experience in a technologically stratified society. As Haraway (5) argued in her foundational political analysis, technological embodiment is never socially neutral but is always embedded within existing structures of power and inequality — an observation that directly informs the distribution of both technology-related risks and access to therapeutic resources.

Paradoxically, vulnerability to technology-related psychological distress may also be unevenly distributed, with marginalized populations potentially facing greater exposure to predatory design practices and exploitative technological systems. Studies by Crawford et al. (30) suggest that body-tracking technologies often employ design features that may disproportionately impact vulnerable individuals, while lacking the regulatory oversight or ethical constraints that might protect users from harm. This creates a troubling situation where those with the least resources may face the greatest risks from technological embodiment while having the least access to protective measures or therapeutic interventions.

Digital literacy represents another crucial dimension of technological embodiment inequality. Work by Eubanks (121) demonstrates how technological literacy intersects with other forms of privilege, creating unequal capacities for critical engagement with body technologies. Individuals lacking sufficient digital literacy may be unable to make informed decisions about technological embodiment while being particularly vulnerable to manipulative or exploitative design practices. This digital divide in embodied technological engagement creates what Lupton (7) describes as differential access to enhancement but also differential vulnerability to novel forms of suffering, requiring targeted interventions that address both technological access and critical digital literacy.

These inequalities have profound implications for clinical practice. Clinicians must recognize that technology-related psychological distress occurs within social contexts that shape both vulnerability and resilience, requiring treatment approaches that address systemic as well as individual factors.

### 6.2 Normalization and pathologization of bodily experience

Body-technology interfaces are actively reshaping cultural definitions of normal and pathological bodily experience, creating new standards and expectations that influence both individual self-perception and clinical practice. This transformation operates through multiple mechanisms that collectively alter the baseline against which embodied experience is evaluated, raising questions about the social construction of bodily normality in the digital era (31, 33).

Digital representations of bodies, particularly through social media platforms and appearance-modification applications, establish new visual norms that become internalized as standards of bodily normality. Research by Tiggemann and Zaccardo (69) has documented how exposure to digitally modified body images shifts perceptions of what constitutes normal bodily appearance, often in directions that are biologically unattainable without technological intervention. This creates a situation where technological mediation becomes necessary to achieve socially constructed norms of bodily acceptability, potentially pathologizing previously normal variations in embodied experience.

Self-tracking and quantification technologies introduce additional layers of normalization by establishing metric-based standards for bodily function and optimization. As Crawford et al. (30) observe, the parameters embedded in self-tracking devices become de facto standards against which bodily experience is evaluated, often without adequate consideration of individual variation or contextual factors. This quantified approach to bodily normality may marginalize forms of embodied knowledge that cannot be easily measured or standardized, while promoting technological dependence for basic self-assessment and regulation.

Virtual and augmented reality technologies further complicate questions of bodily normality by expanding the range of possible embodied experiences beyond biological constraints. King and Warren (83) reveals how virtual embodiment can transform understandings of what constitutes normal bodily capability and experience, potentially creating dissatisfaction with biological limitations while promoting unrealistic expectations for physical embodiment. This expansion of embodied possibilities, while potentially liberating in some contexts, may also contribute to pathologization of natural bodily limitations and variation.

These transformations require ongoing critical reflection on the processes through which bodily normality and pathology are constructed and legitimated in technological contexts. As Canguilhem (33) argued, categories of normality and pathology are always historically situated and value-laden rather than purely objective or scientific. In the rapidly evolving landscape of technological embodiment, maintaining awareness of these constructive processes becomes essential for avoiding inappropriate pathologization while remaining responsive to genuine forms of technological distress.

### 6.3 Informed consent and bodily autonomy

The integration of technology into embodied experience raises complex questions about informed consent and bodily autonomy that challenge traditional frameworks for protecting individual rights and promoting self-determination. These challenges arise from the often invisible or poorly understood ways in which technologies modify embodied experience, the long-term effects of technological embodiment, and the complex interactions between individual choice and technological design (119).

Data collection through tracking devices and other body-technology interfaces creates new forms of surveillance and potential exploitation that individuals may not fully comprehend when adopting these technologies. As van Dijck (122) has demonstrated, the “datafication” of embodied experience transforms intimate bodily information into commercial assets while often obscuring the implications of data sharing and analysis. This raises questions about whether meaningful informed consent is possible when the full implications of technological embodiment cannot be adequately understood or communicated at the time of adoption.

Virtual and augmented embodiment experiences present additional challenges for informed consent by potentially influencing perception, behavior, and identity in ways that may extend beyond the immediate technological encounter. Research by Serino et al. (123) documented changes in body size estimation immediately following a single VR exposure session. It should be noted, however, that this study provides no follow-up data demonstrating persistence beyond the immediate post-VR period, and that most virtual embodiment research documents effects lasting only up to one week without repeated interventions. The informed consent implications therefore concern the immediate and short-term effects of virtual embodiment rather than lasting or irreversible changes to body perception.

Invasive body-machine interfaces, including implantable devices and other forms of technological integration, challenge traditional conceptions of bodily integrity and autonomy by creating hybrid forms of embodiment that blur boundaries between self and technology. As Clark (42) argues, these technologies raise questions about the relationship between technological enhancement and personal identity, requiring new frameworks for understanding autonomy in contexts of technological embodiment.

Algorithmic processing of bodily data introduces additional concerns about autonomy by potentially perpetuating biases and influencing health decisions in ways that may not be transparent or subject to individual control. Studies by Noble (124) have documented how algorithmic systems can reproduce existing social biases while claiming objectivity, potentially undermining individual autonomy in bodily decision-making through apparently neutral technological mediation.

These challenges require the development of new ethical frameworks that can address the novel features of technological embodiment while maintaining core commitments to human dignity and self-determination. As Floridi (125) argues, traditional ethical approaches may be insufficient for addressing the complexities of technologically mediated embodiment, necessitating innovative frameworks that recognize the fundamentally hybrid nature of contemporary embodied experience.

## 7 Future perspectives: toward a techno-corporeal phenomenology

The rapid evolution of body-technology interfaces demands theoretical innovation that can adequately capture the emerging realities of technologically mediated embodiment. The conceptual frameworks that guided understanding of embodied experience in pre-digital contexts require reconsideration to address the novel characteristics of contemporary techno-corporeal existence. This theoretical development must proceed alongside clinical and ethical innovation to ensure that our understanding keeps pace with technological change while remaining grounded in lived experience (6, 60, 61, 119).

### 7.1 Beyond digital/physical dualism

Traditional conceptual frameworks that maintain strict distinctions between “real” and “virtual,” “natural” and “technological,” or “physical” and “digital” embodied experience prove increasingly inadequate for understanding contemporary technological embodiment. As Hansen (126) argues in his analysis of digital embodiment, these binary frameworks fail to capture the complex interpenetration of physical and digital dimensions that characterizes lived technological experience. A contemporary techno-corporeal phenomenology must develop more nuanced conceptual tools that can address the hybrid nature of technological embodiment without losing sight of its existential significance.

The phenomenological tradition, particularly Merleau-Ponty (10) analysis of the body schema as fundamentally open to technological incorporation, provides crucial insights for developing post-dualistic frameworks. Ihde (6) concept of “embodiment relations” demonstrates how technologies become phenomenologically transparent when successfully incorporated into embodied experience, suggesting continuity between mediated and non-mediated perception rather than categorical distinction. This phenomenological approach recognizes that technological embodiment involves transformation rather than replacement of natural embodied capacities.

Contemporary theoretical developments in posthumanism and cyborg studies offer additional resources for moving beyond dualistic frameworks. Hayles (127) conceptualization of embodiment within a “computational universe” recognizes the inextricable intertwining of biological and technological dimensions in contemporary experience, while Haraway (5) notion of cyborg embodiment challenges the assumption that technology represents something external to natural embodiment. These perspectives suggest that technological mediation may be constitutive of rather than opposed to authentic embodied experience.

This post-dualistic understanding has important implications for conceptualizing technology-related psychopathology. Rather than viewing these conditions as deviations from “natural” embodied experience, they must be understood as specific configurations of techno-corporeal existence that may limit rather than enhance human flourishing. This perspective requires moving beyond simple pro-technology or anti-technology positions to develop more nuanced understandings of how particular technological configurations support or undermine psychological wellbeing within specific contexts and for particular individuals.

### 7.2 Toward a clinic of technologically mediated corporeality

The psychological presentations associated with body-technology interfaces described in this manuscript raise genuine questions about how clinical practice should evolve to address the specific features of technologically mediated distress, regardless of whether these presentations constitute validated independent diagnostic entities. This clinical innovation must integrate technological sophistication with phenomenological sensitivity, recognizing both the transformative potential and the risks associated with technological embodiment (48, 97).

Clinical competence in the techno-corporeal era requires what Krueger and Osler (98) describe as integration of technological and experiential knowledge, moving beyond traditional disciplinary boundaries to develop what Ihde (20) terms “material hermeneutics”—interpretive approaches that can understand the meaning of technological engagement within lived experience. This integration requires clinicians to develop both technical understanding of contemporary technologies and phenomenological sensitivity to how these technologies become meaningful within particular embodied subjectivities.

Perhaps most challenging for contemporary clinical practice is developing approaches that can recognize the dual potential of technologies as both pathogenic and therapeutic. As Riva (95) observes, the same technological features that contribute to distress in some contexts may facilitate healing in others, requiring clinical frameworks that can navigate this paradox with sophistication and nuance. This requires moving beyond simplistic technological determinism to embrace what Haraway (5) calls “situated knowledges”—contextual understandings that evaluate technological engagement in relation to specific embodied subjects and their particular circumstances.

### 7.3 Interdisciplinary research and development of new paradigms

Understanding technology-related embodiment disorders requires interdisciplinary research approaches that can integrate insights from neuroscience, phenomenology, computer science, anthropology, and media studies while maintaining methodological rigor and theoretical coherence. This interdisciplinary integration must move beyond mere multidisciplinary collaboration to achieve genuine theoretical synthesis that can capture the irreducible complexity of technologically mediated embodiment (22, 128).

Neuroscientific research provides essential insights into the biological substrates of technological embodiment. Maister et al. (38) and Blanke et al. (36) offer theoretical and integrative accounts of the neural mechanisms supporting body ownership and self-representation, demonstrating how multisensory integration in regions including the temporo-parietal junction and premotor cortex underlies the sense of body ownership. It should be noted that these are review and theoretical papers rather than empirical neuroimaging studies of technology use, and their relevance here is in establishing the normal neural architecture upon which technology-induced modulations operate, rather than as direct evidence for pathological biomarkers of technology-related disorders.

Phenomenological and philosophical approaches offer complementary perspectives that preserve the first-person dimension of technological embodiment while providing sophisticated conceptual frameworks for understanding these experiences. As Zahavi (129) argues, phenomenological methods provide access to dimensions of experience that cannot be captured through third-person observation alone, making them essential for understanding the subjective dimension of technology-related distress.

Computational and information sciences contribute crucial understanding of how specific technological parameters influence embodied experience, with research by Slater and Sanchez-Vives (130) demonstrating systematic relationships between technical features and phenomenological outcomes. This technical understanding enables more precise identification of technological factors that may contribute to or mitigate psychopathological experiences.

Anthropological and sociological perspectives provide essential contextualization of technological embodiment within broader social and cultural systems, helping avoid reductive individualization of technology-related disorders while illuminating the social dimensions of technological distress. Media and visual culture studies contribute additional insights into how digital representations shape embodied self-understanding and social interaction.

The integration of these diverse perspectives requires new research paradigms that can maintain both theoretical sophistication and empirical rigor. The “4E” approach to cognition — referring to the theoretical framework that understands cognition as embodied (grounded in bodily states and sensorimotor processes), embedded (dependent on environmental context), extended (incorporating external tools and technologies into cognitive processes), and enactive (constituted through active engagement with the world rather than passive representation) — offers one promising integrative framework. Its relevance to technology-mediated embodiment lies specifically in its capacity to conceptualize how digital technologies become incorporated into cognitive and bodily self-experience without reducing this incorporation to either purely neural or purely behavioral accounts (22, 128). Fuchs et al. (131) ecological-phenomenological approach to psychopathology provides a complementary model for synthesizing biological, phenomenological, and sociocultural perspectives, grounding clinical understanding in the circular dynamics between lived body, environment, and intersubjective context. These emerging paradigms recognize that technologically mediated embodiment cannot be adequately understood through any single disciplinary lens while maintaining commitment to scientific precision and clinical relevance.

## 8 The role of clinicians in the era of technologically mediated corporeality

### 8.1 New competencies for mental health professionals

Mental health clinicians confronting new forms of bodily psychopathology must develop specific competencies that transcend traditional clinical training. The rapidly evolving technological landscape demands continuous learning and adaptation from practitioners who wish to effectively address these emerging conditions (49, 97).

Advanced technological literacy represents a requirement for contemporary clinical practice. Clinicians must develop nuanced understanding of virtual reality, augmented reality, wearable devices, and digital platforms—not merely as abstract concepts but as lived technologies with specific experiential implications. As Krueger and Osler (98) argue, this literacy must go beyond superficial familiarity to include embodied knowledge of how these technologies feel from the inside. Practical experience with various technological interfaces allows clinicians to develop the empathic understanding necessary for therapeutic engagement with technologically mediated distress.

Beyond general technological literacy, clinicians must cultivate the ability to assess the impact of specific technologies on individual bodily experience. This assessment requires attunement to the idiosyncratic ways in which technologies become incorporated into particular embodied subjectivities. Merleau-Ponty (10) phenomenological account of the body schema — his description of the body as a dynamic, pre-reflective structure through which we engage with the world, fundamentally open to incorporating tools and technologies into its habitual repertoire — remains relevant here because it provides a conceptual vocabulary for understanding how technologies are not experienced abstractly but always through the lens of an individual’s specific bodily history and habitual engagements. This framework has received empirical support from contemporary neuroscience of body ownership and tool incorporation (8, 36), validating its continued clinical utility. Clinicians must therefore develop what Fuchs (132) calls “embodied empathy”—the capacity to sense how technologies become meaningful within a client’s unique embodied perspective.

Sensitivity to generational differences in digital bodily experience constitutes another crucial clinical competency. Rajanala et al., (133) in *JAMA Facial Plastic Surgery* identified “Snapchat dysmorphia,” highlighting how selfie filters generate unrealistic beauty standards and drive aesthetic procedure demands to match digitally altered images. This foundational article examines the shift toward body dysmorphia driven by the desire to emulate filtered, rather than celebrity, appearances. This generational sensitivity requires what Bourdieu (134) termed a “historical” understanding of embodiment—recognizing how bodily habits and perceptions are shaped by specific technological epochs and developmental timing.

Perhaps most challenging for traditionally trained clinicians is developing the ability to utilize technologies themselves as therapeutic tools. As Riva et al. (97) have demonstrated through their pioneering work in VR therapy, technologies that contribute to distress in certain configurations can facilitate healing when applied with therapeutic intention. This paradoxical potential requires clinicians to develop what Ihde (20) calls “instrumental realism”—a sophisticated understanding of how technologies can be intentionally configured to create specific experiential effects. Training programs developed by Wiederhold and Wiederhold (135) offer promising models for developing these technological therapeutic competencies.

Underpinning all these specific skills must be a critical understanding of the socio-technological systems that influence bodily experience. Clinicians need what Winner (136) called “technological politics”—recognition that technologies are never neutral but always embody specific values and power relations. This critical consciousness allows clinicians to help clients contextualize their individual experiences within broader systemic patterns, potentially transforming personal distress into political awareness and collective action. The work of Lupton (7) on critical digital health studies provides valuable resources for developing this systemic perspective.

These competencies require specific training programs and continuous updating to keep pace with rapid technological evolution. The development of such programs represents an area of active need within mental health professional education, requiring interdisciplinary collaboration between clinical training institutions, technology researchers, and practicing clinicians to create curricula that overcome the traditional separation between technological and clinical knowledge (137).

### 8.2 Body-technology interfaces: embodiment, digital mediation, and technology-related psychological distress

A preventive perspective requires active collaboration between clinicians and developers of body-technology interfaces, moving beyond reactive responses to technological harm toward proactive engagement in the design process (119, 138). Clinicians can bring psychological expertise to development teams through participatory design methodologies (139), contribute to ethical guidelines addressing informed consent, privacy, and dependence (140, 141), and support the development of early screening tools that identify problematic patterns before they reach clinical thresholds — while remaining vigilant against the risks of surveillance capitalism (142). The field of positive technology (143–145) offers a complementary framework for designing technologies that actively promote rather than merely avoid undermining psychological wellbeing, creating what Nussbaum (146) might call capability-sensitive interfaces. Beyond individual clinical work, mental health professionals have a responsibility to engage in broader advocacy: promoting regulatory frameworks protecting bodily data privacy (142), supporting media literacy initiatives that help young people develop critical awareness of how technologies shape bodily self-perception (147, 148), and advocating for equitable access to both technologies and therapeutic interventions across social divides of race, class, gender, and ability (121, 149). By engaging in public deliberation about the ethical implications of body-technology interfaces (150, 151), clinicians can help shape the sociotechnical conditions that give rise to — or mitigate — individual suffering (31, 152).

## 9 Conclusions

The analysis of body-technology interfaces and emerging forms of psychopathology highlights both risks and opportunities for human experience. The challenge for the future lies neither in demonizing nor uncritically embracing new technologies, but in developing modalities of conscious integration that honor bodily experience in all its dimensions. This balanced approach recognizes what Ihde (20) termed the “ambiguity” of technology—its capacity to simultaneously expand and constrain human possibilities.

This conscious integration requires, first and foremost, a deep understanding of the transformations of bodily experience in the digital era. As Merleau-Ponty (10) observed, the body is not a static object but a dynamic process of engagement with the world. Digital technologies introduce new possibilities for this engagement, restructuring what Husserl (153) called the “lifeworld”—the pre-reflective background against which all experience takes place. Scholarly work across disciplines has begun to map these transformations, but much remains to be understood about how specific technologies reshape structures of embodied experience.

Equally important is the development of conceptual and clinical tools adequate to new forms of psychopathology. Traditional diagnostic frameworks and therapeutic approaches often assume a pre-technological understanding of embodiment that may not capture the distinctive features of conditions like filter dysmorphia or virtual depersonalization. The phenomenological psychopathology tradition pioneered by Jaspers (154) and developed by contemporary scholars like Fuchs et al. (131) offers valuable resources for this conceptual development, emphasizing the importance of understanding altered subjectivity from within. These conceptual innovations must be paired with practical clinical methodologies that can effectively address technologically mediated distress.

An ethical approach that considers both individual and social implications of body-technology interfaces provides another essential dimension of conscious integration. As Varela (155) argued in his work on ethical expertise, ethics cannot be reduced to abstract principles but must be understood as embodied wisdom developed through practice. This ethical wisdom must encompass both individual clinical decisions about technology use and broader societal questions about technological development and regulation. The capability approach developed by Nussbaum (146) offers a promising framework for this ethical reflection, focusing on how technologies expand or constrain human capabilities for flourishing.

Finally, conscious integration requires interdisciplinary dialogue involving clinicians, researchers, developers, and users of technologies. Each of these perspectives offers essential insights into the complex phenomenon of technological embodiment. The transdisciplinary approach advocated by Nicolescu (156) provides a methodology for this dialogue, creating what Galison (157) calls “trading zones” where different disciplinary languages can find points of productive exchange. Through this collaborative process, we can develop more sophisticated understanding of how technologies shape embodied experience and more effective approaches to promoting wellbeing in a technologically mediated world. Particular attention should be directed toward adolescents and young adults, who represent a population of heightened developmental vulnerability in the context of body-technology interfaces. This life period is characterized by ongoing body image formation, identity consolidation, heightened reward sensitivity, and intensified social comparison processes — all of which may amplify susceptibility to the proposed constructs described in this paper. The normative developmental tasks of adolescence and emerging adulthood intersect with the specific affordances of social media filters, avatar-based gaming, and self-tracking applications in ways that may lower the threshold for clinically relevant presentations. Future research should therefore prioritize developmental samples and longitudinal designs capable of capturing the trajectory from normative digital engagement to pathological presentations across this critical period.

Only through this integrated approach will it be possible to effectively address the challenges posed by the rapid evolution of body-technology interfaces, promoting a bodily experience that—even in its increasing technological hybridization—remains a source of wellbeing and existential meaning. As Merleau-Ponty (10) reminds us, the body is our primary means of “being-in-the-world”—our mode of engagement with existence. The technological mediation of this engagement represents one of the most significant transformations of human experience in our time, demanding thoughtful, nuanced, and ethically informed responses from all who care about human flourishing.
